# The Procoagulant Activity of Apoptotic Cells Is Mediated by Interaction with Factor XII

**DOI:** 10.3389/fimmu.2017.01188

**Published:** 2017-09-25

**Authors:** Aizhen Yang, Fengwu Chen, Chao He, Junsong Zhou, Yi Lu, Jihong Dai, Raymond B. Birge, Yi Wu

**Affiliations:** ^1^Cyrus Tang Hematology Center, Collaborative Innovation Center of Hematology, Soochow University, Suzhou, China; ^2^The Sol Sherry Thrombosis Research Center, Temple University School of Medicine, Philadelphia, PA, United States; ^3^Wuhan Thalys Medical Technology Inc., Wuhan, China; ^4^Department of Pathology and Laboratory Medicine, Rutgers University-New Jersey Medical School, Newark, NJ, United States; ^5^Department of Microbiology, Biochemistry and Molecular Genetics, Rutgers University-New Jersey Medical School, Newark, NJ, United States

**Keywords:** apoptotic cells, factor XII, phosphatidylserine, coagulation, contact activation system

## Abstract

Apoptotic cells, by externalizing phosphatidylserine (PS) as a hallmark feature, are procoagulant. However, the mechanism by which apoptotic cells activate coagulation system remains unknown. Intrinsic coagulation pathway is initiated by coagulation factor XII (FXII) of contact activation system. The purpose of this study was to determine whether FXII is involved in procoagulant activity of apoptotic cells. Using western blotting and chromogenic substrate assay, we found that incubation with apoptotic cells, but not with viable cells, resulted in rapid cleavage and activation of FXII in the presence of prekallikrein and high molecular weight kininogen (HK), other two components of contact activation system. As detected by flow cytometry, FXII bound to apoptotic cells in a concentration-dependent manner, which was inhibited by annexin V and PS liposome. Direct association of FXII with PS was confirmed in a surface plasmon resonance assay. Clotting time of FXII-deficient plasma induced by apoptotic cells was significantly prolonged, which was fully reversed by replenishment with FXII. Corn trypsin inhibitor, a FXII inhibitor, completely prevented apoptotic cells-induced intrinsic tenase complex formation. Consistently, apoptotic cells significantly increased thrombin production in normal plasma, which was not affected by an inhibitory anti-tissue factor antibody. However, blocking of PS by annexin V, inhibition of FXII, or the deficiency of FXII suppressed apoptotic cells-induced thrombin generation. Addition of purified FXII to FXII-deficient plasma recovered thrombin generation to the normal plasma level. In conclusion, FXII binds to apoptotic cells *via* PS and becomes activated, thereby constituting a novel mechanism mediating the procoagulant activity of apoptotic cells.

## Introduction

Apoptosis, or programmed cell death, under physiologic conditions is an active process that is morphologically and biochemically different from necrosis. Apoptosis can be induced in a variety of pathological disorders, including inflammation, autoimmune diseases, atherosclerosis, tissue injury, and degeneration, as well as during radiation treatment and chemotherapy (1). When a cell undergoes apoptosis, phosphatidylserine (PS) typically becomes exposed on the cell membrane (2). If apoptotic cells are not rapidly cleared, they become procoagulant and are often associated with thrombotic disorders, such as atherothrombosis and Trousseau syndrome (3–5). Up to date, the mechanisms by which apoptotic cells activate the coagulation system and enhance blood clotting are largely unknown. Tissue factor (TF) is complexed with circulating coagulation factor VII and triggers the cascade that generates thrombin. Although TF is involved in the procoagulant activity of apoptotic cells induced by inflammatory mediators, such as LPS, only a portion of the thrombin generated during apoptosis is attributable to TF (3, 6, 7), suggesting the existence of additional mechanisms.

The plasma contact activation system, also called the intrinsic coagulation pathway, consists of factor XII (FXII), prekallikrein (PK), and high-molecular-weight kininogen (HK) (8, 9). FXII, or Hageman factor, is a zymogen of the serine protease factor XIIa (FXIIa). FXII zymogen is activated by limited proteolysis, involving cleavage of the Arg353-Val354 peptide bond, generating the two-chain molecule FXIIa (10). Two principal modes of FXII activation exist. In the first, FXII is activated by binding to negatively charged surfaces, which induces a conformational change (auto-activation). In the second, other proteases, such as kallikrein, cleave and activate FXII (hetero-activation) (10). FXIIa consists of a heavy chain and a light chain connected by a single disulfide bond between two cysteine residues (Cys340 and Cys367) (10, 11). For the last four decades, it has been known that FXII is activated by a variety of artificial and biological anionic surfaces (11, 12). These observations suggest that the contact activation system plays an important role in the enhancement of coagulation. However, whether FXII is involved in apoptotic cell-mediated procoagulant activity has never been studied.

In this study, we investigated whether FXII participates in the procoagulant activity of apoptotic cells. Our results indicate that FXII binds to apoptotic cells and rapidly becomes cleaved and activated. The binding of FXII to apoptotic cells is mediated by PS, and activation of FXII is required for apoptotic cell-mediated blood clotting and thrombin formation. Our study reveals a novel function for FXII and a new mechanism underlying apoptotic cell-mediated procoagulant activity.

## Materials and Methods

### Materials

Human FXII, PK, HK, FIX, FX, and FXI were purchased from Enzyme Research Laboratories (South Bend, IN, USA). Recombinant human FVIII was obtained from American Diagnostica, Inc. The EZ-Link^®^ Sulfo-NHS-LC-Biotinylation kit was purchased from Thermo Scientific, Inc. Chromogenic substrate for FXIIa and tissue factor blocking antibody (4501) were purchased from American Diagnostica. The chromogenic substrate for FXa was obtained from Chromogenix. Monoclonal antibody against FXII heavy chain (B7C9) was purchased from Santa Cruz Biotechnology. Monoclonal antibody against FXII light chain (C6B7) and PE-labeled anti-TF antibody were obtained from eBioscience. Polyclonal anti-HK heavy chain antibody was purchased from Abgent. Polyclonal anti-pKal antibody was obtained from Abcam (catalog number 43084). Corn trypsin inhibitor (CTI) was purchased from Merck Chemicals, Ltd. FXII-deficient plasma and pooled human normal plasma were obtained from George King Bio-Medical, Inc. Phosphatidylcholine (PC), PS, 1,2-dioleoyl-sn-glycero-3-phosphocholine (DOPC), and 1-oleoyl-2-{6-[(7-nitro-2-1,3-benzoxadiazol-4-yl)amino]hexanoyl}-sn-glycero-3-phosphocholine (NBD-PC) were obtained from Avanti-Polar Lipids. The FITC-labeled Annexin V Apoptosis Detection kit was purchased from BD Biosciences. Magnetic annexin V microbeads were obtained from Miltenyi Biotech, Inc. Annexin V was obtained from BD PharMingen. Calf intestinal alkaline phosphatase (CIAP) and DNase were purchased from New England Biolabs. RPMI 1640 medium and fetal bovine serum (FBS) were purchased from Invitrogen. All other reagents were obtained from Sigma-Aldrich unless otherwise specified.

### Cell Culture and Induction of Apoptosis

The human T lymphoblastoid cell line CCRF-CEM was purchased from American Type Culture Collection (ATCC^®^ Number: CCL-119™). CEM cells were propagated in complete RPMI-1640 medium supplemented with 10% FBS, 1 mM l-glutamine, and 1% penicillin-streptomycin at 37°C in a humidified atmosphere with 5% CO_2_ (13). To induce apoptosis, cells (2 × 10^5^/mL) were cultured with 10 µM dexamethasone at 37°C in a humidified atmosphere containing 5% CO_2_ for 24 h. Apoptosis was verified by the FITC Annexin V Apoptosis Detection kit according to the manufacturer’s protocol (14). Apoptotic cells were purified with annexin V magnetic microbeads.

### Preparation of Liposomes and Phospholipid-Coated Beads

To prepare liposomes containing 100% PC (PC liposomes) or PC and PS at 80:20 mol% (PS liposomes), the appropriate amounts of each phospholipid were dissolved with chloroform in a glass tube. Before use, the phospholipids were dried under nitrogen, suspended in PBS, and sonicated for 3 min (15, 16). The phospholipid concentration was determined by a phosphorus assay. To prepare phospholipid-coated beads, 6.5 mg/mL Nucleosil 120-3 C18 beads (Macherey-Nagel) in 1 mL of chloroform were incubated with 2.5 mg/mL DOPC (PC beads) or mixed DOPC/PS (80:20 mol%; PS beads). The phospholipid-coated beads were dried under nitrogen, resuspended in PBS, sonicated, and then labeled with 2.5 µM NBD-PC for 20 min at 37°C. After centrifugation at 13,000 × *g* for 10 min, the beads were washed with ice-cold PBS twice to remove remaining NBD-PC, and then resuspended in PBS.

### Flow Cytometric Analysis of FXII Binding to Apoptotic Cells

Human FXII was biotinylated using the EZ-Link^®^ Sulfo-NHS-LC-Biotinylation Kit according to the manufacturer’s protocol. Biotin-labeled FXII (B-FXII) at various concentrations was incubated with apoptotic cells at 4°C for 15 min. After washing, the cells were labeled with PE-avidin and analyzed by flow cytometry.

### Measurement of FXII Activity and Cleavage

Cells were incubated with FXII in the presence or absence of PK and HK in HEPES buffer (137 mM NaCl, 5 mM HEPES, 2.7 mM KCl, 2 mM MgCl_2_, 0.42 mM NaH_2_PO_4_, and 1% BSA, pH 7.5) supplemented with 50 µM ZnCl_2_ at 37°C for 30 min. After centrifugation at 2,500 rpm for 5 min, the supernatant was collected and a chromogenic substrate, Pefachrome FXIIa (0.5 mM), was added. The optical density of substrate hydrolysis was measured at 405 nm using a spectrophotometer (SpectraMax M5) (17). Cleavage of FXII was also detected by western blotting with a monoclonal antibody against FXII heavy chain. The density of the bands was measured by NIH Image J, and cleavage was defined as ratio of the percentage of cleaved FXIIa (48 kDa)/[uncleaved FXII (80 kDa) + cleaved FXIIa (48 kDa)].

### Surface Plasmon Resonance Assay

The experiments were performed at 25°C using HBS-N buffer (20 nM HEPES and 0.15 M NaCl, pH 7.4) containing 50 µM Zn^2+^ as a running buffer. PS liposomes (PS:PC = 1:9) were diluted in running buffer and immobilized onto flow cell 2 (FL2) of the L1-sensor chip, and PC liposomes were immobilized onto flow cell 1 (FL1) of the L1-sensor chip. Subsequent measurements were obtained at a flow rate of 30 µL/min. A two-fold dilution series of FXII diluted in running buffer was generated (0, 2.5, 5, 10, 20, 40, 80, 100, 200, 400 nM) and was injected over the flow cells at a flow rate of 10 µL/min for 120 s and dissociated for 300 s in order of increasing concentration. Response to PS liposome binding curves was obtained by subtracting the FL2 curve from the FL1 curve and analysis using BIAevaluation software.

### Intrinsic Tenase Complex Activity

Apoptotic cells were incubated with 200 µL of HEPES buffer containing 2.5 mM CaCl_2_, 95 nM purified human FXII, 30 nM PK, 30 nM HK, 5.8 nM purified human FXI and FIX, 0.25 nM purified human FVIII, and (where indicated) 2 µM CTI at 37°C. Then, the reaction was started by the addition of purified human FX (170 nM). At various time points, a 25-µL aliquot of the mixture was removed, and 5 µL of 60 mM EDTA in PBS was added to stop FXa formation. FXa formation was monitored as the hydrolysis of the chromogenic substrate S-2222 (0.2 mM) over 30 min. Optical density at 405 nM was converted to FXa nM using a dilution curve of human FXa.

### Clotting Time Assay

Blood drawn from drug-free healthy volunteers was anticoagulated by adding 1 part sodium citrate (110 mM) to 9 parts whole blood. Our study using blood from healthy volunteers was performed after approval by the IRBs of Temple University (IRB no. 20857) and Soochow University (IRB no. 2012037), obtaining informed consent, and in accordance with the Declaration of Helsinki. Platelet-poor plasma (PPP) was prepared by centrifugation at 2,000 × *g* for 30 min. In some experiments, commercial FXII-depleted plasma and normal plasma were used to examine the role of FXII in apoptotic cell-mediated clotting and thrombin generation. After viable or apoptotic cells were mixed with plasma and incubated at 37°C for 180 s, 50 µL of warmed 20 mM CaCl_2_ was added to start the reaction, and clotting time was immediately recorded with an Amelung KC4A coagulometer (18).

### Thrombin Generation Assay

Thrombin generation in plasma was measured over time with a coagulation analyzer Ceveron^®^ alpha (Technoclone, Vienna, Austria), which employs a fluorogenic thrombin substrate (Z-Gly-Gly-Arg-AMC) to continuously monitor thrombin activity in plasma. Measurements were conducted in a total volume of 150 µL, including 40 µL of normal plasma or FXII-deficient plasma. Apoptotic cells were pretreated with 5 µg/mL BSA, annexin V, mouse IgG, and anti-TF antibody (4501) as indicated. The plasma was treated with mouse IgG and anti-FXII antibody (C6B7) when there was a need to block FXII. Then, an 80-µL aliquot of cells (1 × 10^5^) was added to a 40-µL plasma sample. After incubation at 37°C for 15 min, 30 µL of fluorogenic buffer (2.5 mM fluorogenic substrate and 87 mM CaCl_2_) was added to start the thrombin generation assay.

### SDS-PAGE and Western Blotting

Cleavage of FXII into FXIIa was detected by SDS-PAGE (12%) under reducing conditions and immunoblotting. After incubation with apoptotic or viable cells as described above, the cells were pelleted by centrifugation, and samples containing FXII were collected (14). These samples and samples from a cell-free system were mixed with SDS-PAGE loading buffer and heated at 95°C. After the samples were separated by electrophoresis and transferred to a polyvinylidene difluoride membrane (Millipore), the membrane was blocked with 5% non-fat dry milk in blocking buffer. After extensive washing, the immunoblots were incubated for 2 h with the primary antibodies, including monoclonal anti-FXII (B7C9), polyclonal anti-HK heavy chain, and polyclonal anti-pKal antibodies. Antibody binding was detected by IRDye 800-conjugated goat anti-mouse IgG (LI-COR Bioscience) or IRDye 680-conjugated goat anti-rabbit IgG (LI-COR Bioscience) and visualized with the ODYSSEY infrared imaging system (LI-COR).

### Identification of TF Antigen by Flow Cytometry

Cells (1 × 10^6^/mL) were incubated with PE-conjugated monoclonal TF (CD142) antibody or isotype control IgG1 for 30 min at 4°C in the dark. Cells were resuspended in 400 µL of PBS before analysis. The mean fluorescence intensity of 10,000 events was determined for each sample.

### Statistical Analysis

The results are expressed as the mean ± SEM of at least three experiments. One-way analysis of variance followed by Tukey’s test (for multiple groups) or Student’s *t*-test (for comparisons between two groups) was used, and a *p* value less than 0.05 was considered statistically significant. Unless stated otherwise, the data shown are from a single experiment that is representative of at least three separate experiments.

## Results

### FXII Binds to Apoptotic Cells *via* Phosphatidylserine (PS)

**Figure 1:**
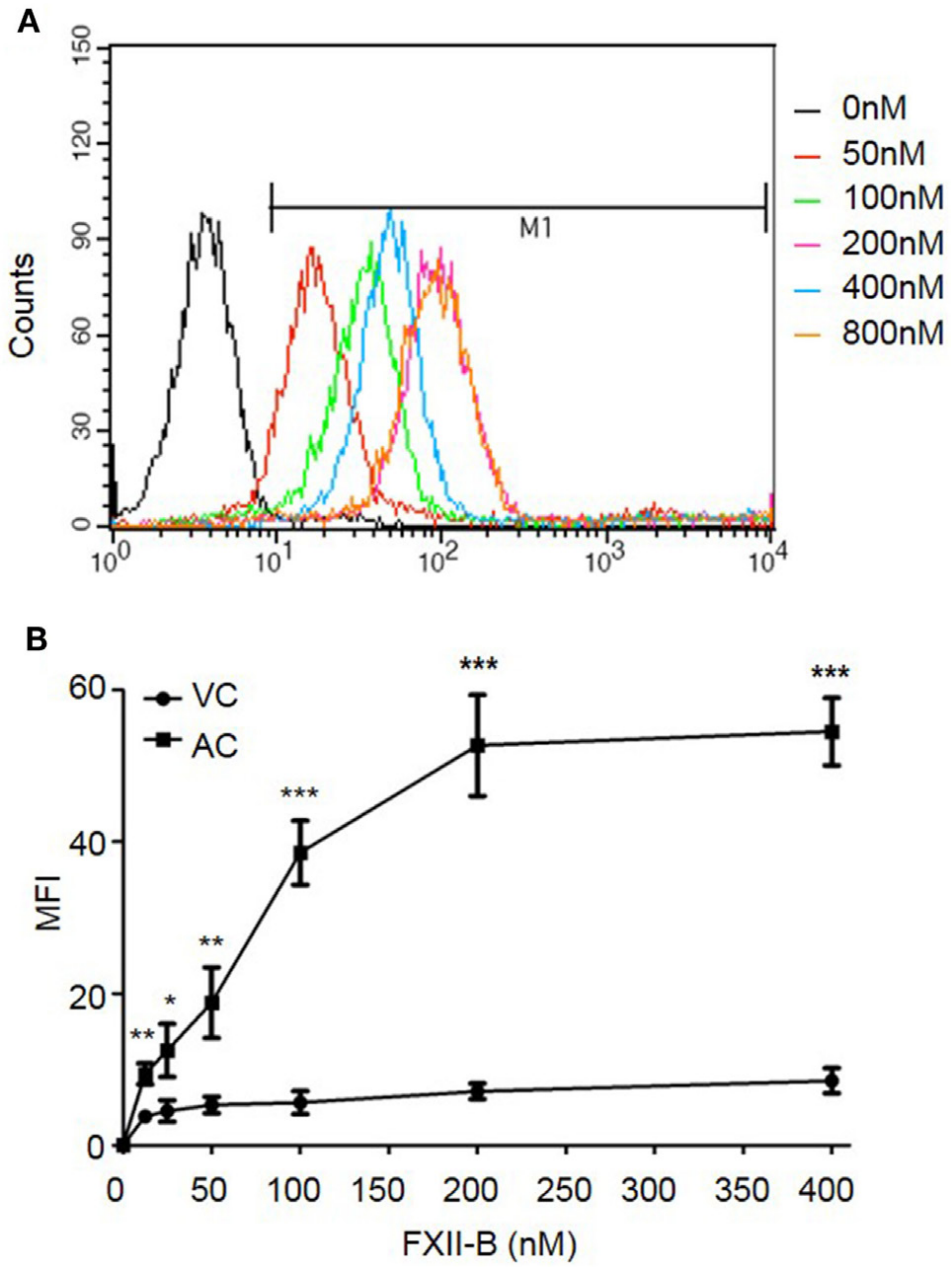
FXII preferentially binds to apoptotic cells. **(A)** Biotin-labeled FXII (B-FXII) at the indicated concentrations was incubated with apoptotic CEM cells (2 × 10^5^) at 4°C for 15 min. After washing, the cells were labeled with PE-avidin and analyzed by flow cytometry. The fluorescence tracings shown are representative data from three independent experiments. **(B)** Viable CEM cells (VC, closed circles) or apoptotic CEM cells (AC, squares) were incubated with B-FXII at the indicated concentrations at 4°C for 15 min. After washing, the cells were labeled with PE-avidin and analyzed by flow cytometry (*n* = 5). The data were analyzed by Student’s *t-*test, *p < 0.05; **p < 0.01; ***p < 0.001.

**Figure 2:**
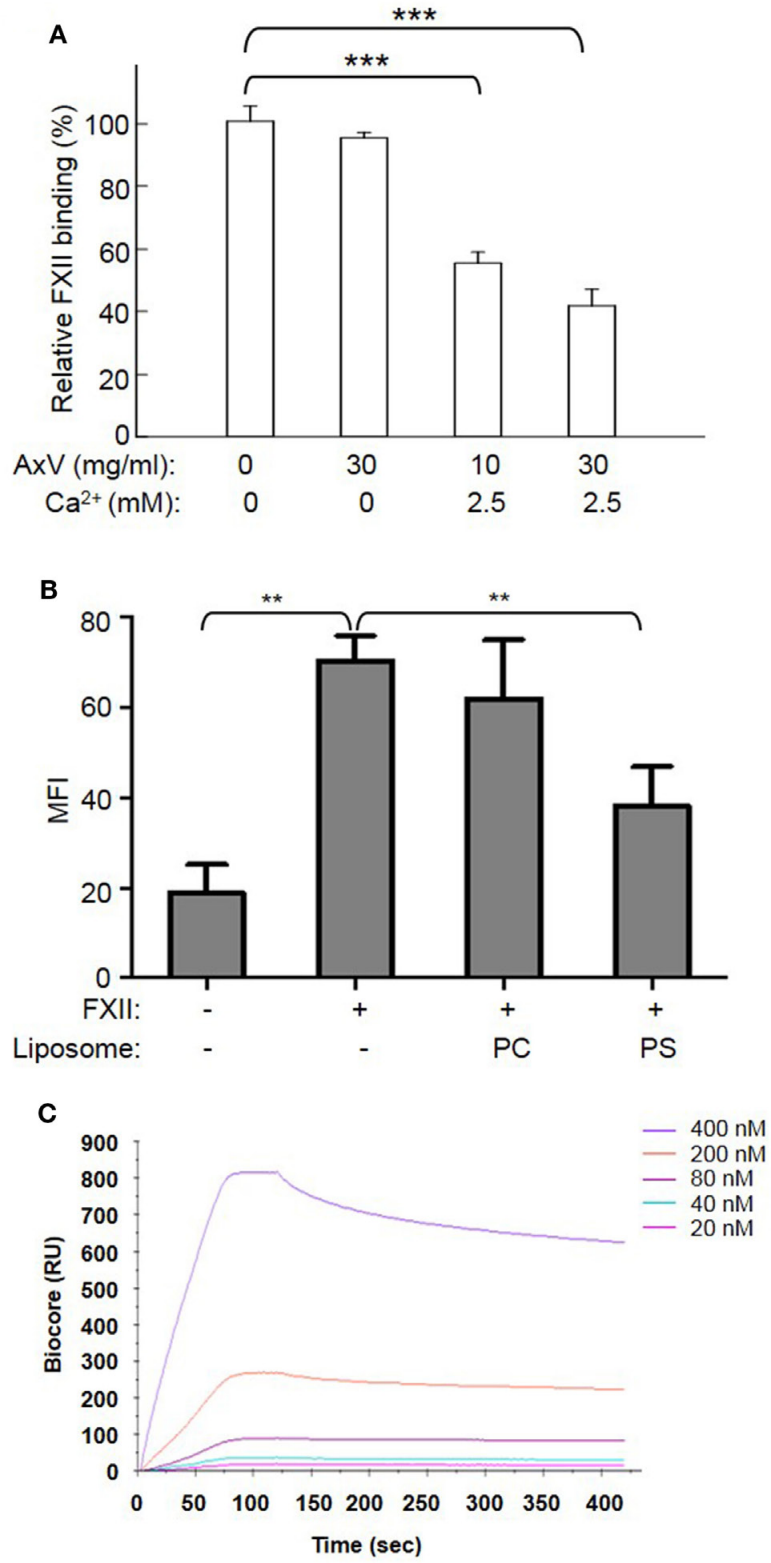
Binding of FXII to apoptotic cells is dependent on phosphatidylserine (PS). **(A)** After preincubation with or without annexin V at the indicated concentrations in the presence of 2.5 mM CaCl_2_ for 20 min, apoptotic CEM cells were labeled with 100 nM B-FXII and PE-avidin as described above. The binding of FXII was analyzed by flow cytometry and indicated as a percentage compared to the binding in the absence of annexin V and CaCl_2_, which was set to 100% (*n* = 4). ***p < 0.001. **(B)** B-FXII (100 nM) was preincubated with or without 1 mM phosphatidylcholine (PC) or PS liposomes at 4°C for 15 min, and then apoptotic cells were added. After incubation for 60 min, the binding of B-FXII to apoptotic cells was analyzed by flow cytometry as described above, and shown as the mean fluorescence intensity (MFI). **p < 0.01. **(C)** FXII binds to phosphatidylserine in a surface plasmon resonance assay. Serial concentrations of FXII were flowed over PS or PC liposomes immobilized on the Biacore sensor chip. The response curve of PS/FXII binding was obtained by subtracting the curve of PC from that of PS. Curves were analyzed with BIAevaluation software. The *K*_D_ was 3.857E-9 M, and the *R*_max_ was 787.0 Ru.

To determine whether FXII binds to apoptotic cells, apoptotic cells were incubated with B-FXII. As detected by flow cytometry, the binding of B-FXII to apoptotic cells increased as the concentration increased (from 50 to 800 nM), and became saturated at 400 nM (Figures 1A,B). However, B-FXII did not bind to viable cells when added at the same concentrations (Figure 1B), suggesting that FXII specifically binds to apoptotic cells. PS is a phospholipid component that is usually maintained on the inner-leaflet (the cytosolic side) of the cell membrane by flippase (2). However, when a cell undergoes apoptosis, PS becomes exposed on the surface of the cell (2). Annexin V (or Annexin A5) is a member of the annexin family of intracellular proteins that binds to PS in a calcium-dependent manner. To examine whether FXII binds to apoptotic cells through PS, apoptotic cells were preincubated with annexin V or BSA in the presence or absence of 2.5 mM CaCl_2_. As shown in Figure 2A, preincubation with annexin V markedly inhibited the binding of FXII to apoptotic cells in the presence of 2.5 mM CaCl_2_, and its inhibitory effect was concentration dependent. In contrast, no inhibition by annexin V was observed in the absence of CaCl_2_ (Figure 2A), suggesting that the inhibitory effect of annexin V is specific. In addition, when FXII was preincubated with PS liposomes, its binding to apoptotic cells was significantly reduced by more than 55% (Figure 2B). However, preincubation of FXII with PC liposomes did not affect binding to apoptotic cells (Figure 2B). To evaluate the binding capacity of FXII to PS liposomes, a Biacore assay was used. The sensorgrams showed an increase in response units that was reflective of PS binding, and the binding response was concentration dependent, with a *K*_D_ of 3.857E-9 M (Figure 2C). These results suggest that FXII binds to apoptotic cells through a high-affinity association with PS.

### The Binding of FXII to Apoptotic Cells Leads to Its Cleavage and Activation

**Figure 3:**
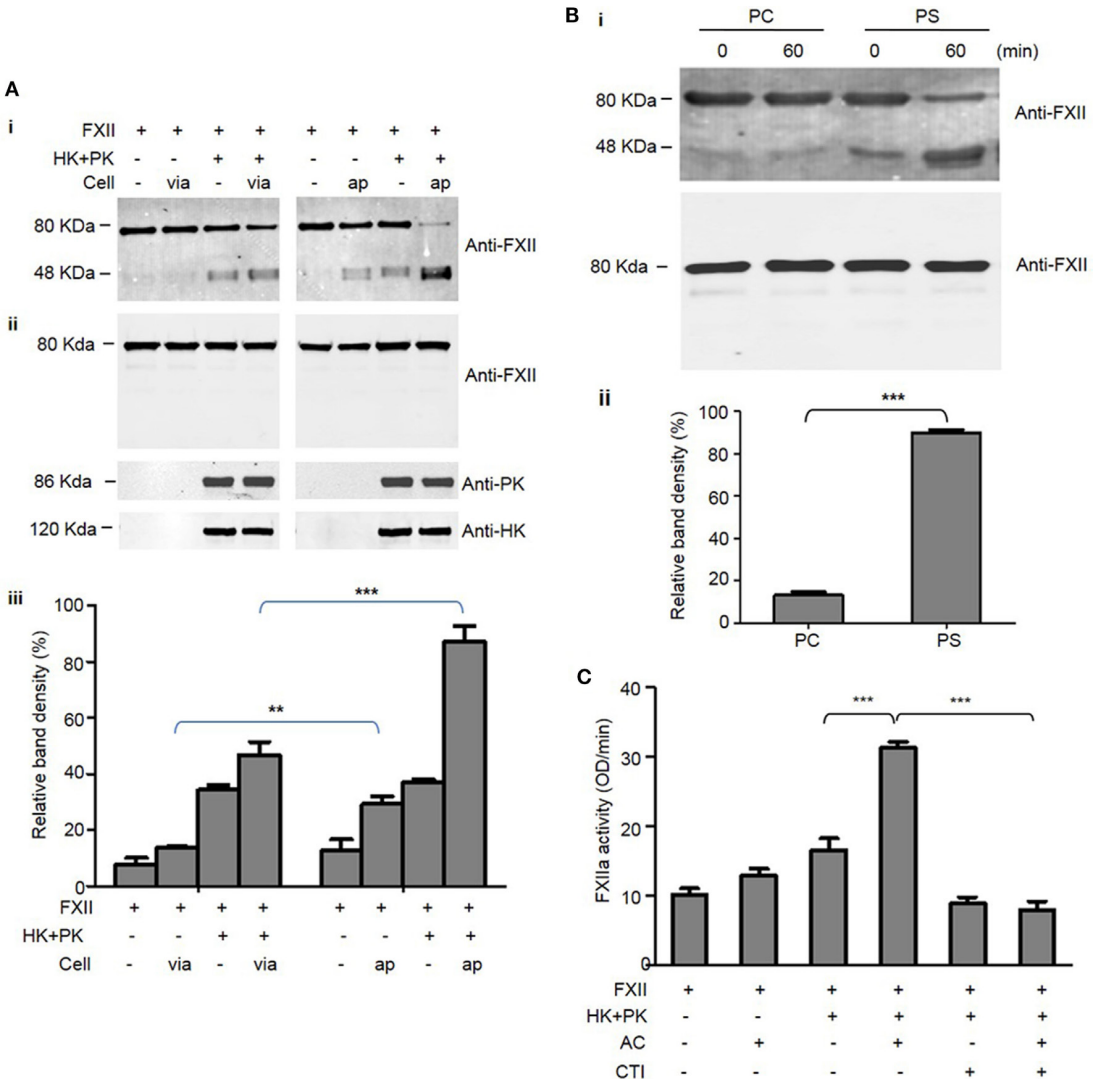
Binding of FXII to apoptotic cells mediates its cleavage and activation. **(A)** As indicated, 95 nM FXII was incubated with viable cells (*via*) or apoptotic cells (ap) at density of 2 × 10^5^ in the presence or absence of 30 nM prekallikrein (PK) and 30 nM high molecular weight kininogen (HK) at 37°C for 30 min. After centrifugation at 2,500 rpm for 5 min, the supernatant was collected and analyzed by western blot with an anti-FXII Ab (i). The levels of FXII, PK, and HK before incubation with cells are shown by western blotting (ii). In three independent experiments, the density of bands was measured by NIH Image J software, and the cleavage of FXII was defined as the ratio of [cleaved FXII chain (48 kDa)]/[uncleaved FXII (80 kDa) plus cleaved FXII chain (48 kDa)] shown as relative band density (iii). **p < 0.01; ***p < 0.001. **(B)** In a cell-free system, 95 nM FXII was mixed with 30 nM PK and 30 nM HK followed by addition of 50 nM phosphatidylserine (PS) or phosphatidylcholine (PC) liposomes, one half of the mixture was immediately centrifuged (0 min), and the other half was incubated at 37°C for 60 min (60 min). After centrifugation at 55,000 rpm for 5 h, the supernatant was collected for analysis by western blotting using anti-FXII Ab (i, upper panel). The levels of FXII before incubation with liposomes are shown by western blotting (i, lower panel). In three independent experiments, the density of bands was measured, and the cleavage of FXII was calculated as described above and shown as relative band density (ii). ***p < 0.001. **(C)** Binding of FXII to apoptotic cells mediates its activation. As indicated, FXII (95 nM) was incubated with or without apoptotic cells (AC; 2 × 10^5^), PK (30 nM) plus HK (30 nM), and corn trypsin inhibitor (CTI) (2 µM) at 37°C for 30 min. After centrifugation at 2,500 rpm for 5 min, the supernatant was collected and factor XIIa (FXIIa) activity was analyzed as the hydrolysis of a chromogenic substrate as described in the Section “Materials and Methods” (*n* = 3). ***p < 0.001.

We next examined whether FXII binding to apoptotic cells induces its cleavage and activation. FXII is converted to its active form, FXIIa, through auto-activation induced by contact with charged surfaces. Thus, the activation of FXII is dependent on its cleavage (8). As shown by western blotting using an antibody recognizing the heavy chain of FXII (Figure 3A, i and ii), incubation with apoptotic cells markedly induced the cleavage of full-length FXII zymogen (80 kDa) to produce a heavy chain fragment (48 kDa). However, FXII was not cleaved when incubated with viable cells (Figure 3A). More strikingly, the FXII cleavage induced by contact with apoptotic cells was significantly enhanced in the presence of HK and PK (Figure 3A). In the absence of cells, incubation of FXII with HK and PK induced cleavage of a small percentage of FXII (Figure 3A), suggesting that FXII may form a complex with HK and PK on the surface of test tubes, thus leading to its cleavage. As shown by densitometric measurement of band intensity, about 85% of the FXII was cleaved in the presence of HK, PK, and apoptotic cells, which was significantly higher than that induced in the presence of viable cells (Figure 3A, iii). Consistent with the observations in the presence of apoptotic cells, incubation with PS liposomes induced cleavage of >80% of the added FXII (Figure 3B, i and ii). In contrast, <15% of the FXII was cleaved when incubated with PC liposomes (PS liposome vs. PC liposomes, *p* < 0.001; Figure 3B). Taken together, PS-mediated FXII binding to apoptotic cells results in its cleavage. To further examine whether the cleaved FXII induced by apoptotic cells is proteolytically active, we performed an assay with a chromogenic substrate. As shown in Figure 3C, in the presence of HK and PK, incubation of FXII with apoptotic cells significantly increased FXIIa activity (*p* < 0.001). This increase in activity was completely prevented by the addition of a FXII inhibitor, corn trypsin inhibitor (CTI). This result suggests that binding of FXII to apoptotic cells promotes its activation.

### FXII Is Required for Apoptotic Cell-Mediated Intrinsic Tenase Formation and Procoagulant Activity

**Figure 4:**
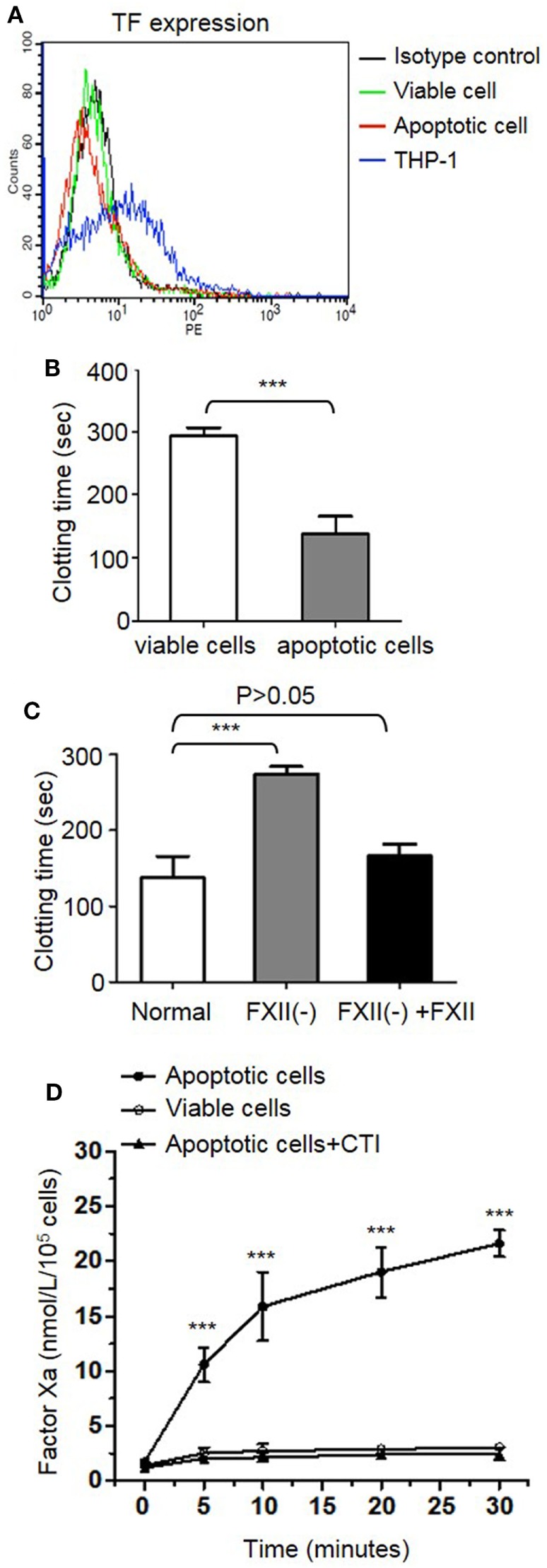
Apoptotic cell-mediated procoagulant activity and intrinsic tenase formation is dependent on FXII activation. **(A)** Tissue factor (TF) expression as determined by flow cytometry. CEM cells treated with (apoptotic cells) or without (viable cells) 10 µM dexamethasone for 24 h were stained with CD142-PE to analyze TF expression on the cell surface. THP-1 cells were used as a positive control. The background staining with an isotype control is shown in black. **(B)** After viable cells and apoptotic cells (2 × 10^5^) were suspended in 150 µL of platelet-poor plasma, clotting was triggered by the addition of 20 mM CaCl_2_ and measured as described in the Section “Materials and Methods” (*n* = 3). ***p < 0.001. **(C)** After apoptotic cells (2 × 10^5^) were suspended in normal plasma (normal), FXII-deficient plasma [FXII(-)] and FXII-deficient plasma supplemented with 375 nM FXII [FXII(-) + FXII], respectively, clotting was triggered by the addition of 20 mM CaCl_2_ and measured as described in the Section “Materials and Methods” (*n* = 3). **(D)** Effect of apoptotic cells on intrinsic tenase complex formation. As indicated, apoptotic or viable cells were incubated with FXII, prekallikrein, high molecular weight kininogen, FXI, FIX, and FVIII. Then FX was added, and tenase complex formation was analyzed using a chromogenic substrate (*n* = 3). Some samples were also treated with 2 µM corn trypsin inhibitor (CTI). ***p < 0.001.

The observation that apoptotic cells can activate FXII led us to hypothesize that this FXII activation is involved in apoptotic cell-mediated procoagulant activity. First, we determined whether the extrinsic coagulation pathway is involved in apoptotic cell-mediated procoagulant activity in our assay system. As evaluated by FACS analysis (Figure 4A), TF antigen remained undetectable on both viable and apoptotic CEMs. THP-1 served as a TF-positive control. Apoptotic CEM-mediated pro-coagulation is not dependent on the extrinsic coagulation pathway, which allowed us to evaluate the role of FXII in this process. In the clotting assay, the addition of apoptotic cells significantly shortened the clotting time of PPP triggered by the addition of CaCl_2_, compared with the clotting time for viable cells (151.8 ± 2.1 vs. 281.9 ± 2.5 s, *p* < 0.001; Figure 4B). To evaluate the contribution of FXII, we measured the clotting time of PPP with apoptotic cells in the presence and absence of FXII. As shown in Figure 4C, compared to the clotting time of normal plasma with apoptotic cells, the clotting time of FXII-deficient plasma was significantly longer (150.8 ± 3.7 vs. 281.4 ± 7.9 s; *p* < 0.001), which was reversed by replenishment with a physiological concentration of purified FXII protein (165.7 ± 6.6 s), suggesting the requirement of FXII for apoptotic cell-mediated pro-coagulation. We next tested the capacity of FXII to initiate tenase formation, which contributes to the procoagulant activity of apoptotic cells. As shown in Figure 4D, in the presence of FXII, PK/HK, FIX, and FVIII, apoptotic cells increased the activity of FXa as a function of time, which was 7.3-fold higher than that with viable cells at 30 s. To examine whether apoptotic cell-mediated FX activation was FXII-dependent, we tested the inhibitory effect of CTI and found that 2 µM CTI reduced apoptotic cell-mediated FXa activation to basal levels (Figure 4D). The above results demonstrate that FXII is critical for intrinsic tenase complex formation, contributing to the procoagulant activity of apoptotic cells.

### FXII Is Important for Apoptotic Cell-Induced Thrombin Generation

**Figure 5:**
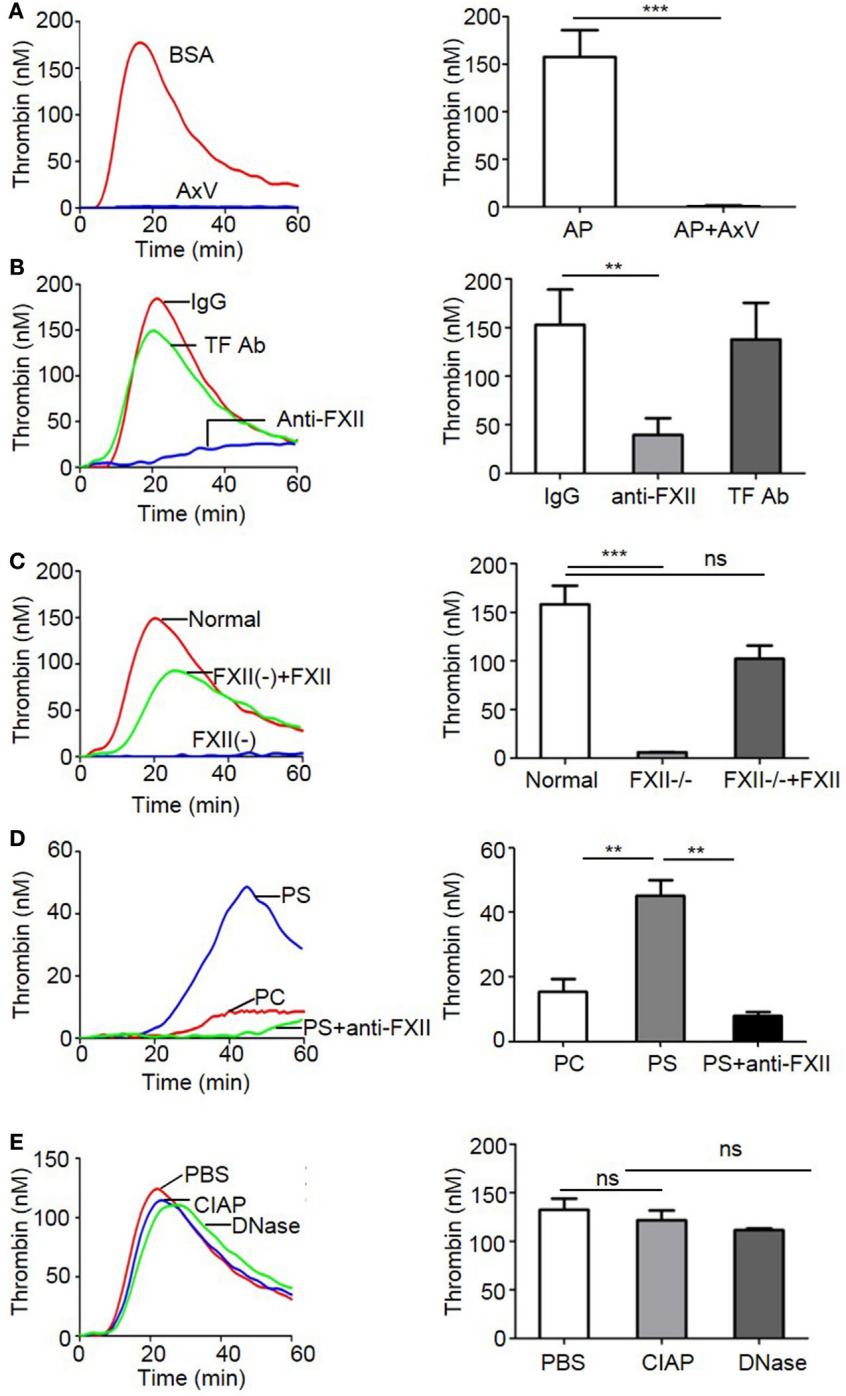
FXII is important in thrombin generation induced by apoptotic cells. **(A–E)** Thrombin generation triggered by apoptotic cells in normal plasma was analyzed as described in the Section “Materials and Methods.” Representative data are shown on the left, and accumulated data with statistical analysis are shown on the right. **(A)** Apoptotic cells were pretreated with 5 µg/mL BSA or annexin V, and then used in a thrombin generation assay (*n* = 3). **(B)** Apoptotic cells were pretreated with an anti-FXII antibody C6B7 or anti-tissue factor (TF) blocking antibody (4501; 5 µg/mL each), and then used in a thrombin generation assay (*n* = 3). **(C)** The thrombin generation assay was performed in normal plasma, FXII-deficient plasma, and FXII-deficient plasma reconstituted with human FXII (*n* = 3). **(D)** Phosphatidylcholine (PC) or phosphatidylserine (PS) liposome-coated beads were used to stimulate thrombin generation in normal plasma. Some samples using PS liposome-coated beads were also incubated with 5 µg/mL C6B7 (*n* = 3). **(E)** Apoptotic cells pretreated with PBS, 100 U/mL Calf intestinal alkaline phosphatase (CIAP), or 0.01% DNase, before thrombin generation was measured. **p < 0.01; ***p < 0.001.

To further determine the contribution of PS to the FXII-dependent procoagulant activity of apoptotic cells, we employed a thrombin generation assay. As shown in Figure 5A, apoptotic cells dynamically induced thrombin generation, which was almost entirely inhibited by annexin V (AxV). Importantly, an inhibitory anti-FXII antibody (C6B7) greatly decreased apoptotic cell-mediated thrombin generation, whereas an anti-TF blocking antibody (TF Ab) did not have such an effect (Figure 5B). Consistently, apoptotic cells failed to induce thrombin generation in FXII-deficient plasma. However, the addition of 375 nM purified FXII to FXII-deficient plasma increased thrombin generation (Figure 5C). To determine the effect of PS on thrombin generation, PS or PC liposome-coated beads were used in a test of thrombin generation activity. As shown in Figure 5D, PS liposomes strongly induced thrombin generation, whereas PC liposomes had only a minor effect. Interestingly, the inhibitory anti-FXII antibody C6B7 greatly diminished PS-induced thrombin generation (Figure 5D), suggesting that FXII contributes to apoptotic cell-mediated thrombin generation by interacting with PS. It is important to note that there is no clear distinction between apoptosis and other cell types of cell death, such as necrosis, and all dying cells exhibit increased PS exposure and enhanced coagulation. Nucleic acids and polyphosphate that are released from necrotic cells can be also associated with FXII activation (19, 20). However, after pretreatment with CIAP and DNase, which degrade polyphosphate and nucleic acids, respectively, there was no change in the level of thrombin generation induced by apoptotic cells (Figure 5E), indicating that polyphosphates and nucleic acids are not involved in the observed apoptotic cell-mediated thrombin generation.

## Discussion

Changes in phospholipid asymmetry, with outer surface exposure of PS, is a fundamental feature of apoptosis. On activated platelets, exposure of PS on the outer leaflet is essential for membrane assembly of the coagulation factor complexes, including the tenase complex, which are necessary for thrombin generation (21). Similarly, upon apoptotic cell death, PS is exposed on the membrane surface; concomitantly, they become procoagulant and activate the coagulation pathway. These enhanced procoagulant activities associated with membrane PS exposure have been widely observed in apoptotic endothelial cells, vascular smooth muscle cells, lymphocytes, monocytes, and cancer cells. It has been shown that tissue factor does not play a major role in this process, as there is no increase in antigen levels or the functional activity of tissue factor (3). Thus, the connection between apoptotic cells and coagulation was largely unknown. In this study, we showed that the FXII zymogen preferentially binds to apoptotic cells, leading to its rapid cleavage and activation, thereby contributing to apoptotic cell procoagulant activity.

Thrombin generation is initiated by two distinct pathways, and it can be triggered by exposure of blood to either a damaged vessel wall (extrinsic) or blood-borne factors (intrinsic). The intrinsic pathway of coagulation is initiated by FXII in a reaction involving HK and PK. These factors are collectively referred to as the contact activation system. The present findings of a correlation between thrombin generation and apoptosis showed increased intrinsic tenase activity; however, there is no available model that outlines how thrombin formation could be initiated by highly negatively charged cellular surfaces when functional TF is absent. It is known that FXII is activated by a variety of artificial or biological anionic surfaces, such as kaolin (22), ellagic acid (23), polymers (24), nucleotides (25), sulfatides (26), glycosaminoglycans (27), misfolded proteins (28), polyphosphates (29), and collagen (30). In this study, we showed, for the first time, that FXII zymogen directly binds to PS liposomes and PS on apoptotic cells (Figures 1 and 2). The binding of FXII to PS mediates its rapid cleavage and activation in the presence of HK and PK (Figure 3). These results suggest that PS on apoptotic cells serves as a docking site for FXII binding, which may induce its auto-activation. The PS on apoptotic cells may also recruit HK and PK to form a complex with FXII, thereby activating PK, and activated PK may increase FXII activation. In three assays, including APTT, intrinsic tenase formation, and thrombin generation assays, we employed FXII-deficient plasma and FXII inhibitor and provided evidence that FXII plays an important role in apoptotic cell-mediated coagulation. We further showed that TF is not involved in the FXII-mediated procoagulant activity of apoptotic cells, as a TF Ab did not affect thrombin generation by apoptotic cells, which is consistent with the absence of TF expression on the surface of apoptotic cells (Figure 4A). In another study, we found that HK binds to apoptotic cells *via* PS, leading to cleavage and the production of bradykinin in the presence of FXII and PK (31). Therefore, PS on apoptotic cells may serve as a novel activator of FXII and a docking site for the assembly of the contact activation system, which may account for the role of FXII in apoptotic cell-mediated procoagulant activity.

This new function of FXII in apoptotic cell-mediated coagulation provides novel insight into the pathology of apoptotic cells. In pathological settings, such as autoimmune disease, chemotherapy, and inflammation, numerous cells frequently undergo apoptosis. Rapid clearance of these apoptotic cells is crucial for maintaining an anti-inflammatory and antithrombotic state (2). However, if these apoptotic cells are not removed efficiently, they may become procoagulant and proinflammatory. For example, in patients with acute leukemia, phagocytes are overwhelmed by the large numbers of apoptotic cells because of uncontrolled leukemic cell proliferation and cytotoxic chemotherapy. In patients with leukemia, coagulation parameters could be upregulated by chemotherapy; however, this cannot account for the hypercoagulable state, as the basal levels of contact activation system components, including FXII, are fairly high (>50 μg/mL). Patients with systemic lupus erythematosus (SLE) often develop microvessel thrombi, concomitant with the accumulation of apoptotic cells (5). The enhanced functional interaction of apoptotic cells with activation of coagulation system likely plays a major role. Kunzelmann et al. (32) demonstrated that malignant hematopoietic cells (HEL cells) trigger blood coagulation through phosphatidylserine exposure (32). When apoptosis occurs in a microenvironment in direct contact with circulating coagulation factors such as FXII, they may contribute to the initiation and enhancement of unique pro-coagulants on the apoptotic cell surface (33). In view of the increasing evidence for the occurrence of vascular cell apoptosis in the above pathological settings, it is important to characterize the mechanism underlying FXII-driven contact system activation when vascular cells become apoptotic. Because FXII can also activate plasminogen in the fibrinolytic pathway (34), how activated FXII integrates the intrinsic coagulation and fibrinolysis systems on the surface of apoptotic cells is an interesting topic for future investigation.

In conclusion, the current study demonstrates that FXII binds to apoptotic cells *via* PS, leading to activation of FXII. Activated FXII contributes to intrinsic tenase formation and blood clotting. This study not only revealed a novel mechanism underlying apoptotic cell-mediated procoagulant activity but also identified the apoptotic cell membrane as a new activation surface for the assembly and activation of the contact activation system. These findings suggest that the role of the contact activation system in apoptotic cell-related procoagulant events requires further investigation. Moreover, it will be worthwhile to explore whether inhibition of FXII could be a therapeutic strategy for the prevention and treatment of apoptotic cell-associated thrombotic diseases.

## Author Contributions

AY, FC, CH, JZ, and JD performed research, analyzed data. YL contributed critical reagent. RB and YW designed research and interpreted the data. YW wrote the paper.

## Conflict of Interest Statement

The authors declare that the research was conducted in the absence of any commercial or financial relationships that could be construed as a potential conflict of interest.

## References

[1] Fuchs Y Steller H Programmed cell death in animal development and disease. Cell (2011) 147:742–58. 10.1016/j.cell.2011.11.045 22078876 PMC4511103

[2] Wu Y Tibrewal N Birge RB. Phosphatidylserine recognition by phagocytes: a view to a kill. Trends Cell Biol (2006) 16:189–97. 10.1016/j.tcb.2006.02.003 16529932

[3] Bombeli T Karsan A Tait JF Harlan JM. Apoptotic vascular endothelial cells become procoagulant. Blood (1997) 89:2429–42. 9116287

[4] Flynn PD Byrne CD Baglin TP Weissberg PL Bennett MR. Thrombin generation by apoptotic vascular smooth muscle cells. Blood (1997) 89:4378–84. 9192761

[5] Casciola-Rosen L Rosen A Petri M Schlissel M. Surface blebs on apoptotic cells are sites of enhanced procoagulant activity: implications for coagulation events and antigenic spread in systemic lupus erythematosus. Proc Natl Acad Sci U S A (1996) 93:1624–9. 10.1073/pnas.93.4.1624 8643681 PMC39992

[6] Wang J Weiss I Svoboda K Kwaan HC. Thrombogenic role of cells undergoing apoptosis. Br J Haematol (2001) 115:382–91. 10.1046/j.1365-2141.2001.03095.x 11703340

[7] Fu Y Zhou J Li H Cao F Su Y Fan S Daunorubicin induces procoagulant activity of cultured endothelial cells through phosphatidylserine exposure and microparticles release. Thromb Haemost (2010) 104:1235–41. 10.1160/TH10-02-0102 20886178

[8] Colman RW Schmaier AH Contact system: a vascular biology modulator with anticoagulant, profibrinolytic, antiadhesive, and proinflammatory attributes. Blood (1997) 90:3819–43. 9354649

[9] Maas C Renne T. Regulatory mechanisms of the plasma contact system. Thromb Res (2012) 129(Suppl 2):S73–6. 10.1016/j.thromres.2012.02.039 22398015

[10] Björkqvist J Nickel KF Stavrou E Renné T. In vivo activation and functions of the protease factor XII. Thromb Haemost (2014) 112:868–75. 10.1160/TH14-04-0311 25187064

[11] Muller F Renne T. Novel roles for factor XII-driven plasma contact activation system. Curr Opin Hematol (2008) 15:516–21. 10.1097/MOH.0b013e328309ec85 18695377

[12] Renne T Schmaier AH Nickel KF Blomback M Maas C. In vivo roles of factor XII. Blood (2012) 120:4296–303. 10.1182/blood-2012-07-292094 22993391 PMC3507141

[13] Tibrewal N Wu Y D’Mello V Akakura R George TC Varnum B Autophosphorylation docking site Tyr-867 in Mer receptor tyrosine kinase allows for dissociation of multiple signaling pathways for phagocytosis of apoptotic cells and down-modulation of lipopolysaccharide-inducible NF-κB transcriptional activation. J Biol Chem (2008) 283:3618–27. 10.1074/jbc.M706906200 18039660

[14] Wu Y Singh S Georgescu MM Birge RB. A role for Mer tyrosine kinase in alphavbeta5 integrin-mediated phagocytosis of apoptotic cells. J Cell Sci (2005) 118:539–53. 10.1242/jcs.01632 15673687

[15] Yeung T Gilbert GE Shi J Silvius J Kapus A Grinstein S. Membrane phosphatidylserine regulates surface charge and protein localization. Science (2008) 319:210–3. 10.1126/science.1152066 18187657

[16] Martinez J Almendinger J Oberst A Ness R Dillon CP Fitzgerald P Microtubule-associated protein 1 light chain 3 alpha (LC3)-associated phagocytosis is required for the efficient clearance of dead cells. Proc Natl Acad Sci U S A (2011) 108:17396–401. 10.1073/pnas.1113421108 21969579 PMC3198353

[17] van der Meijden PE Munnix IC Auger JM Govers-Riemslag JW Cosemans JM Kuijpers MJ Dual role of collagen in factor XII-dependent thrombus formation. Blood (2009) 114:881–90. 10.1182/blood-2008-07-171066 19372258

[18] Xie R Gao C Li W Zhu J Novakovic V Wang J Phagocytosis by macrophages and endothelial cells inhibits procoagulant and fibrinolytic activity of acute promyelocytic leukemia cells. Blood (2012) 119:2325–34. 10.1182/blood-2011-06-362186 22138513

[19] Geddings JE Mackman N Recently identified factors that regulate hemostasis and thrombosis. Thromb Haemost (2014) 111:570–4. 10.1160/TH13-10-0812 24573314 PMC4080798

[20] Smith SA Morrissey JH Polyphosphate: a novel modulator of hemostasis and thrombosis. Arterioscler Thromb Vasc Biol (2015) 35:1298–305. 10.1161/ATVBAHA.115.301927 25908762 PMC4441552

[21] Schoenwaelder SM Yuan Y Josefsson EC White MJ Yao Y Mason KD Two distinct pathways regulate platelet phosphatidylserine exposure and procoagulant function. Blood (2009) 114:663–6. 10.1182/blood-2009-01-200345 19387006

[22] Colman RW Mattler L Sherry S Studies on the prekallikrein (kallikreinogen) – kallikrein enzyme system of human plasma. II. Evidence relating the kaolin-activated arginine esterase to plasma kallikrein. J Clin Invest (1969) 48:23–32. 10.1172/JCI105971 4237065 PMC322188

[23] Ratnoff OD Crum JD Activation of Hageman factor by solutions of ellagic acid. J Lab Clin Med (1964) 63:359–77. 14165678

[24] Nossel HL Wilner GD LeRoy EC Importances of polar groups for initiating blood coagulation and aggregating platelets. Nature (1969) 221:75–6. 10.1038/221075a0 5782621

[25] Paul A Avci-Adali M Neumann B Guo K Straub A Dietz K Aptamers influence the hemostatic system by activating the intrinsic coagulation pathway in an in vitro Chandler-Loop model. Clin Appl Thromb Hemost (2010) 16:161–9. 10.1177/1076029608329580 19117958

[26] Tans G Griffin JH. Properties of sulfatides in factor-XII-dependent contact activation. Blood (1982) 59:69–75. 6914902

[27] Renne T Schuh K Muller-Esterl W. Local bradykinin formation is controlled by glycosaminoglycans. J Immunol (2005) 175:3377–85. 10.4049/jimmunol.175.5.3377 16116231

[28] Maas C Govers-Riemslag JW Bouma B Schiks B Hazenberg BP Lokhorst HM Misfolded proteins activate factor XII in humans, leading to kallikrein formation without initiating coagulation. J Clin Invest (2008) 118:3208–18. 10.1172/JCI35424 18725990 PMC2518075

[29] Muller F Mutch NJ Schenk WA Smith SA Esterl L Spronk HM Platelet polyphosphates are proinflammatory and procoagulant mediators in vivo. Cell (2009) 139:1143–56. 10.1016/j.cell.2009.11.001 20005807 PMC2796262

[30] Cheng Q Tucker EI Pine MS Sisler I Matafonov A Sun MF A role for factor XIIa-mediated factor XI activation in thrombus formation in vivo. Blood (2010) 116:3981–9. 10.1182/blood-2010-02-270918 20634381 PMC2981546

[31] Yang A Dai J Xie Z Colman RW Wu Q Birge RB High molecular weight kininogen binds phosphatidylserine and opsonizes urokinase plasminogen activator receptor-mediated efferocytosis. J Immunol (2014) 192:4398–408. 10.4049/jimmunol.1302590 24688027 PMC4134950

[32] Kunzelmann C Freyssinet JM Martínez MC. Rho A participates in the regulation of phosphatidylserine-dependent procoagulant activity at the surface of megakaryocytic cells. J Thromb Haemost (2004) 2:644–50. 10.1111/j.1538-7836.2004.00688.x 15102021

[33] Romero-Díaz J García-Sosa I Sánchez-Guerrero J. Thrombosis in systemic lupus erythematosus and other autoimmune diseases of recent onset. J Rheumatol (2009) 36:68–75. 10.3899/jrheum.071244 19012362

[34] Mitchell JL Lionikiene AS Georgiev G Klemmer A Brain C Kim PY Polyphosphate colocalizes with factor XII on platelet-bound fibrin and augments its plasminogen activator activity. Blood (2016) 128:2834–45. 10.1182/blood-2015-10-673285 27694320 PMC5325051

